# Supplementary material for: Complex regulation of Gephyrin splicing is a determinant of inhibitory postsynaptic diversity
Source: Nat Commun. 2022 Jun 18;13:3507. doi: 10.1038/s41467-022-31264-w (PMC9206673; doi:10.1038/s41467-022-31264-w)
Supplement: Supplementary file 1 — Supplementary information [file 41467_2022_31264_MOESM1_ESM.pdf]

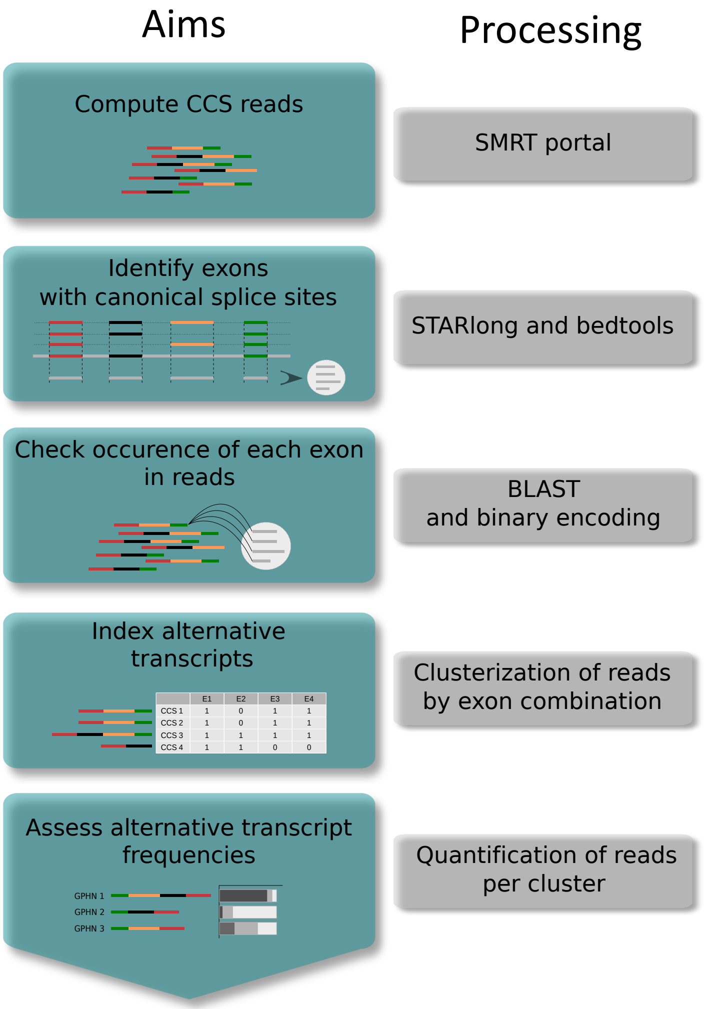

Supplementary Fig.1: Schematic boxes describing the mixed pipeline (informatic and manual) used to analyze Circular Consensus Sequences obtained by PacBio sequencing.

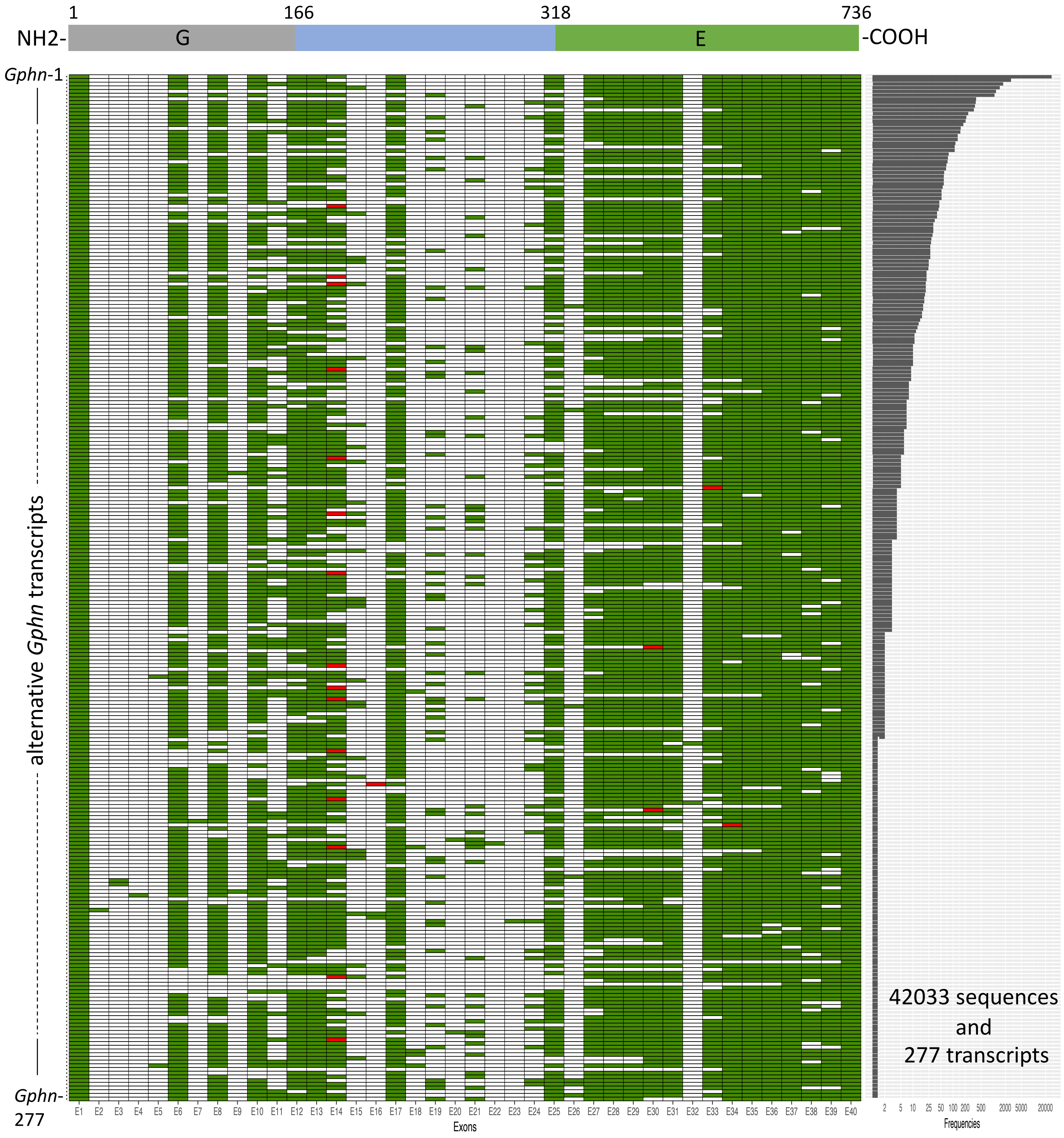

Supplementary Fig.2: *Gphn* alternative transcripts detected by PacBio sequencing.
The 277 alternative *Gphn* transcripts identified from 42033 SMRT sequences, each line represents a distinct transcript and each column symbolizes one exon named at the bottom. Transcripts are ordered and named *Gphn*-1 to *Gphn*-277 for the most to the least detected respectively. Exons are displayed as a rectangle, green for included, white for skipped and red for exon included with an alternative 3’ splice site. The protein domains (G and E) and the central unstructured region (blue) of GPHN are represented at the top. The abundance of each transcript is reported on a bar graph established with a log scale.

exons with alternative 3'ss

40 annotated Gphn exons

		Exon	transcipts distribution
		E1	277
		E2	1
		E3	2
		E4	1
		E5	2
		E6	234
		E7	1
		E8	231
		E9	2
		E10	224

		Exon	transcipts distribution
		E11	62
		E12	227
		E13	236
		E14	173
		E15	30
		E16	2
		E17	230
		E18	5
		E19	52
		E20	54

		Exon	transcipts distribution
		E21	43
		E22	1
		E23	1
		E24	43
		E25	215
		E26	7
		E27	219
		E28	232
		E29	233
		E30	242

		Exons	transcipts distribution
		E31	240
		E32	2
		E33	235
		E34	263
		E35	266
		E36	270
		E37	269
		E38	254
		E39	251
		E40	277

Exons	transcipts distribution
EA	15
EB	1
EC	2
ED	1
EE	1

mm9 / mm10 — Alternative exon annotated in VastDB

Supplementary Fig.3: Frequency of exon inclusion in the 277 *Gphn* alternative transcripts.
Table displaying how many times an exon participates in the 277 alternative transcripts. Alternative exons annotated in VastDB are displayed with blue and green squares depending on mm9 and mm10 genome annotation respectively.

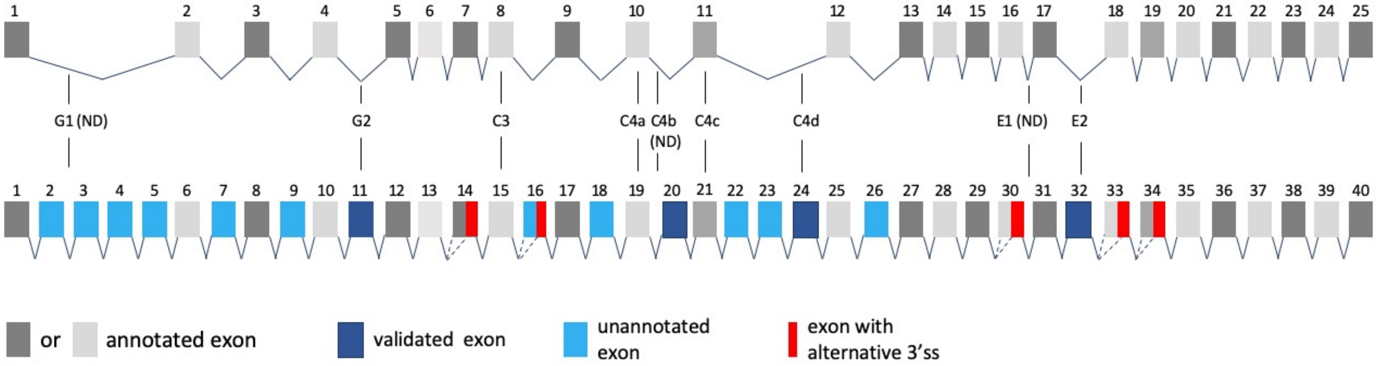

Supplementary Fig.4: Exon architecture of *Gphn*.
Illustrations comparing GPHN exon architecture reported from Ensembl and literature data (top) versus the exon annotation determined in this study (Bottom).

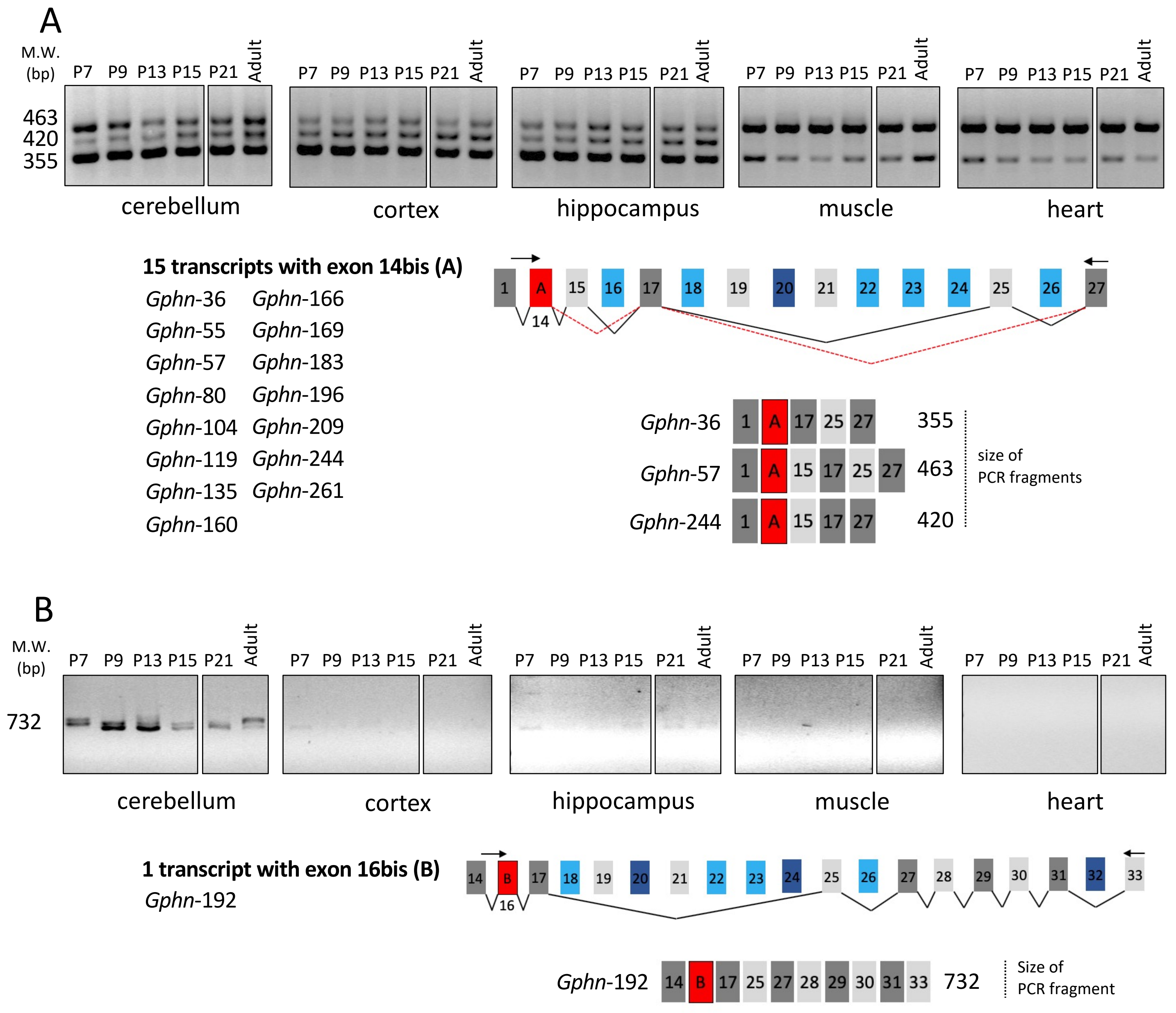

Supplementary Fig.5-6: RT-PCR validation of *Gphn* transcripts.
RT-PCR analysis of transcripts carrying exons spliced with an alternative 3'ss of exon 14 (**A**) and 16 (**B**). The amplified PCR fragment(s) are displayed at the top of each panel in 5 different tissues, at 6 distinct developmental stages, were separated in agarose gel and stained with ethidium bromide. At the bottom right, we show a schematic view of expected PCR fragment(s) and their sizes based on selected primers. At the bottom left, we have listed all *Gphn* alternative transcripts carrying each alternative exon. Samples and RT-PCR analysis were replicated n=2. Source data are provided as a Source Data file.

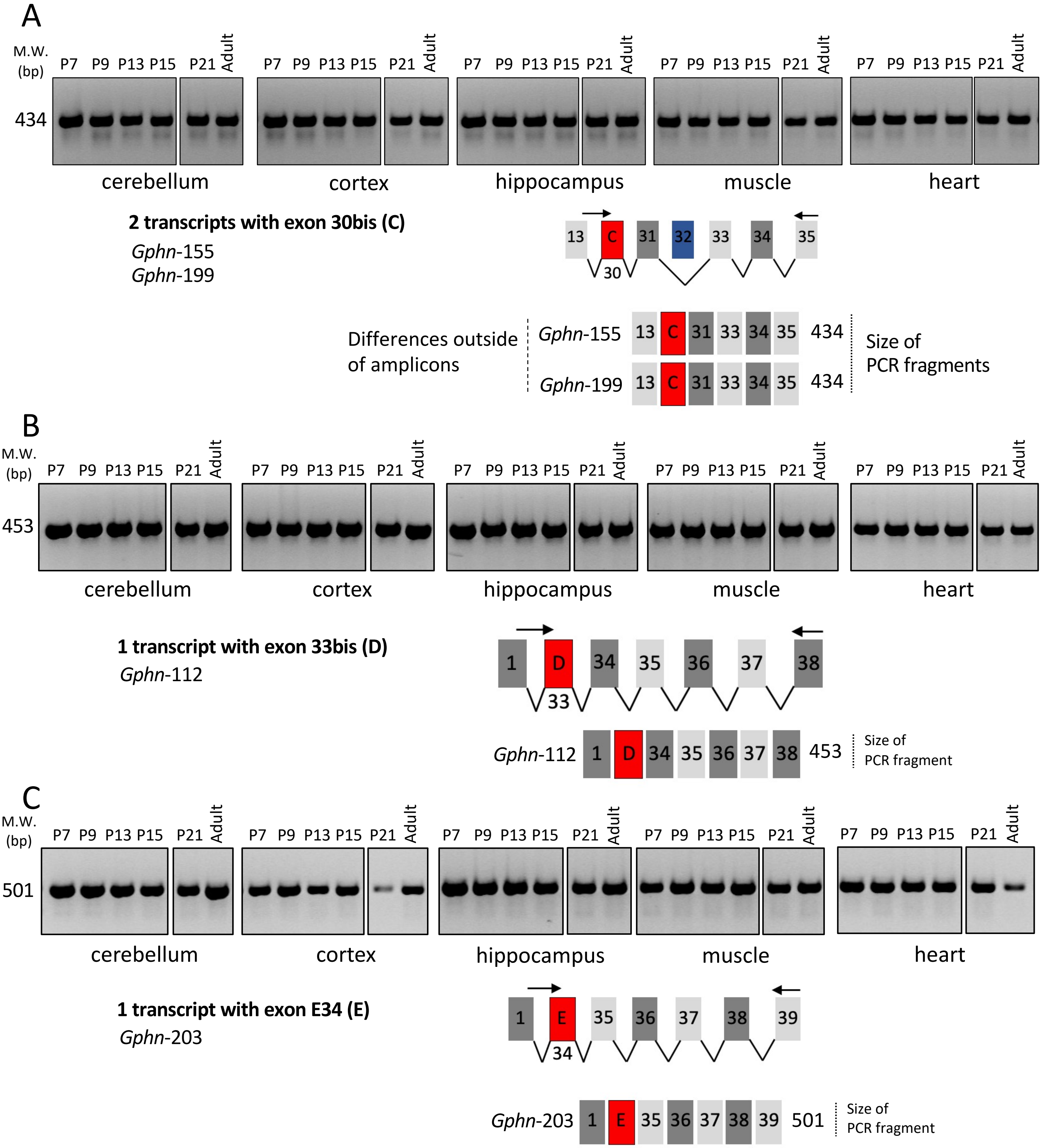

Supplementary Fig.5-6: RT-PCR validation of *Gphn* transcripts.

Analysis by RT-PCR of transcripts carrying exons selected with an alternative 3'ss (exon A, B, C, D, E that correspond to alternative 3'ss of exon, 14, 16, 30, 33 and 34 respectively). At the top of each panel, PCR fragment(s) amplified in 5 different tissues, at 6 distinct developmental stages, were separated in agarose gel and stained with ethidium bromide. At the bottom right, we show a schematic view of expected PCR fragment(s) and their sizes based on selected primers. At the bottom left, we have listed all *Gphn* alternative transcripts carrying each alternative exon. Samples and RT-PCR analysis were replicated n=2. Source data are provided as a Source Data file.

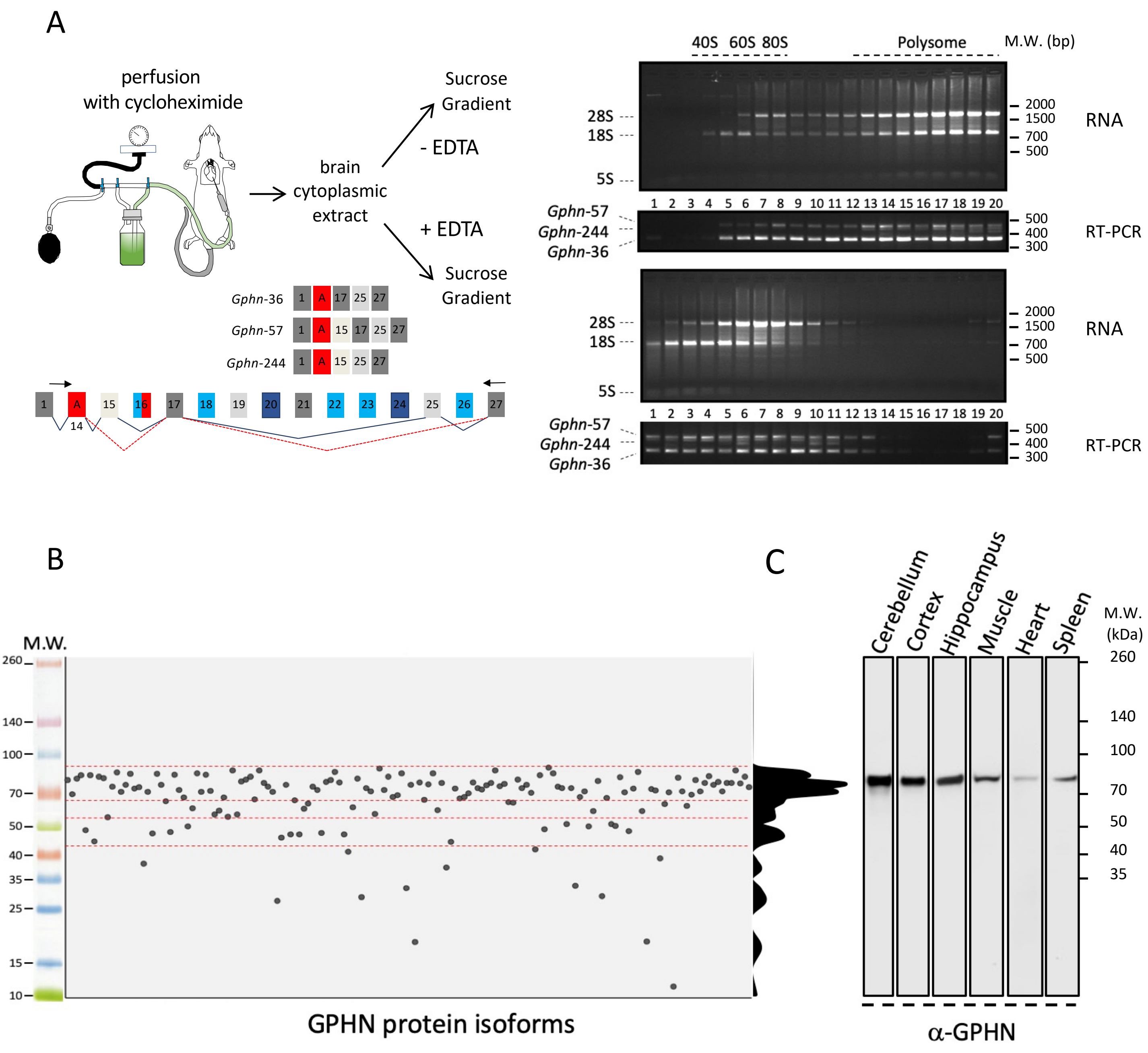

Supplementary Fig.7: *Gphn* transcripts engaged in the translation machinery, and GPHN protein enrichment.

(A) Illustration displaying the procedure used to separate RNA associated with the polyribosome. Brain extracts isolated from mouse perfused with cycloheximide were prepared and separated onto a sucrose gradient to distinguish fractions containing RNA engaged in translation (polyribosome). The treatment of the extract with EDTA dissociates ribosomes and was used as a control. Ribosomal RNA (28S, 18S, 5S) were analyzed in agarose gel for each fraction, while *Gphn* transcripts showed in Supplementary Fig.5A detected using RT-PCR amplification (*Gphn*-57, *Gphn*-244 and *Gphn*-36). The full procedure (perfusion of mouse, sample preparation and RT-PCR) was replicated n=2. (B) In silico PAGE-SDS graph displaying the molecular weight separation associated with the 154 GPHN theoretical isoforms. On the left side, a molecular weight ladder is shown for reference. On the right side, a density graph shows isoform distribution that doesn't consider expression levels of each isoform. (C) Western blot of GPHN isoforms in different mouse tissue using the mab3B11 antibody, this analysis was replicated n=2. Source data are provided as a Source Data file.

A

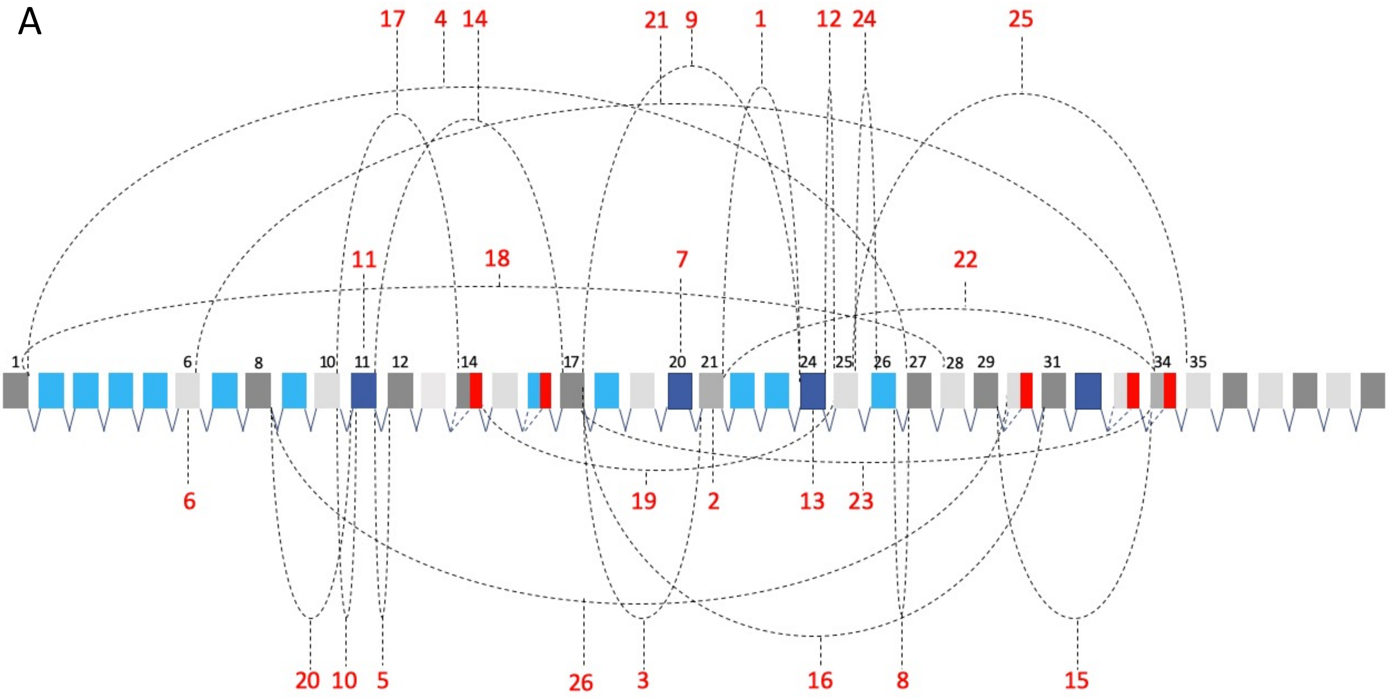

B

		number of isoforms	number of matched peptides			number of isoforms	number of matched peptides
1	EEJ:21-24	11	4	14	EEJ:11-17	2	1
2	Exon 21	11	4	15	EEJ:29-34	2	1
3	EEJ:17-21	13	6	16	EEJ:17-31	1	1
4	EEJ:1-27	12	4	17	EEJ:10-14	1	4
5	EEJ:11-12	37	1	18	EEJ:1-28	2	1
6	Exon 6	1	1	19	EEJ:14-25	7	1
7	Exon 20	39	1	20	EEJ:8-11	4	1
8	EEJ:26-27	7	2	21	EEJ:6-34	1	1
9	EEJ:17-24	10	2	22	EEJ:21-34	1	1
10	EEJ:10-11	57	5	23	EEJ:17-34	11	1
11	Exon 11	57	6	24	EEJ:25-26	7	1
12	EEJ:24-25	41	17	25	EEJ:25-35	2	1
13	Exon 24	43	2	26	EEJ:8-30	1	1

Supplementary Fig.8: Exon-exon junctions corresponding to novel GPHN peptides identified by proteomic.
(A) Cartoon displaying exon-exon junctions (EEJ) or exons corresponding to novel GPHN peptides detected by mass spectrometry. (B) Table summarizing the EEJs and corresponding peptides identified by mass spectrometry. Number of GPHN isoforms containing the corresponding peptides are also indicated.

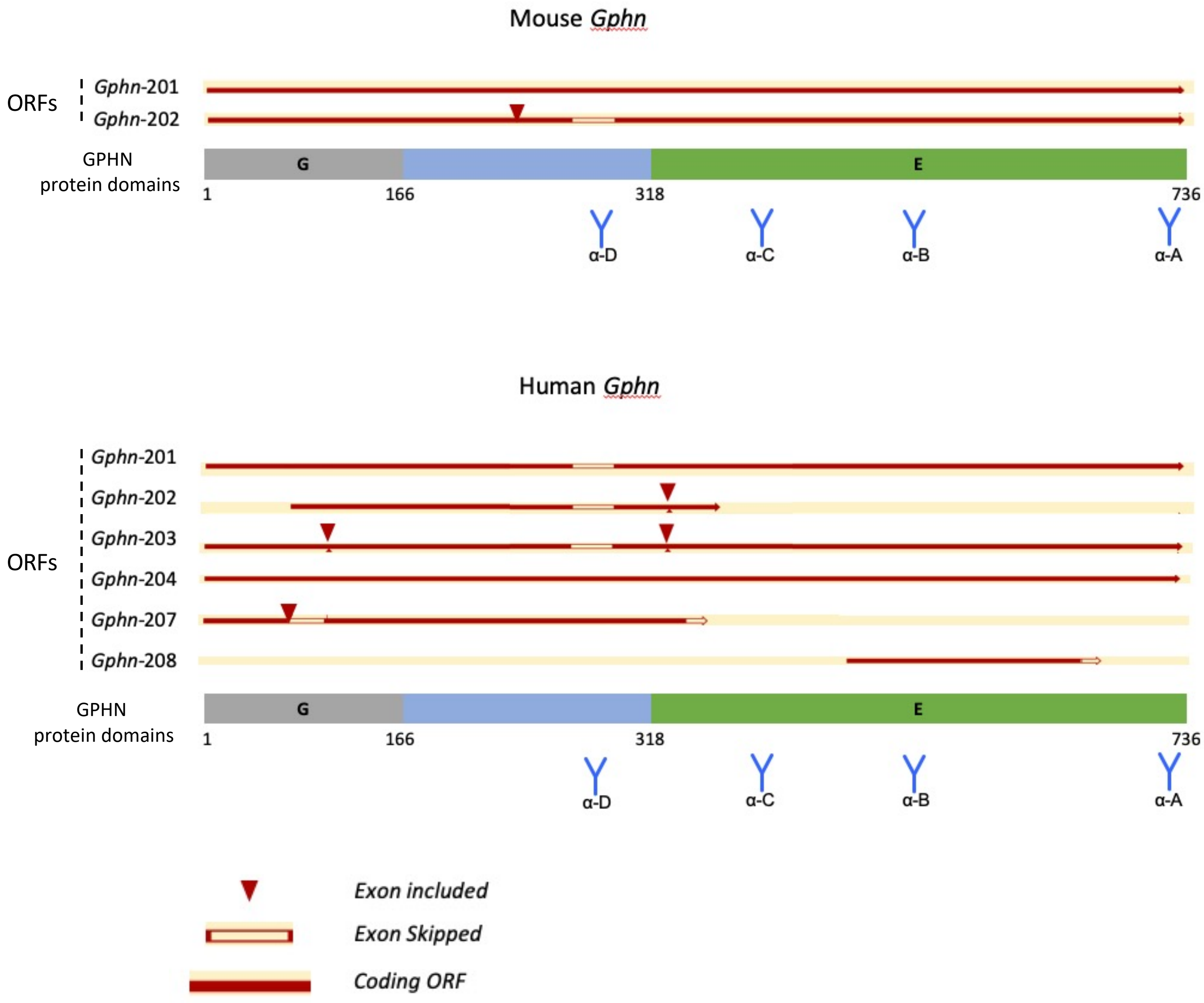

Supplementary Fig.9: Schematic representation of the mouse and human protein domains of GPHN with the mapping of epitopes recognized by antibodies used in this study.
On the top of each drawing, GPHN ORFs translated from *Ensembl* ESTs are displayed with the positioning of exon inclusion and exon skipping.

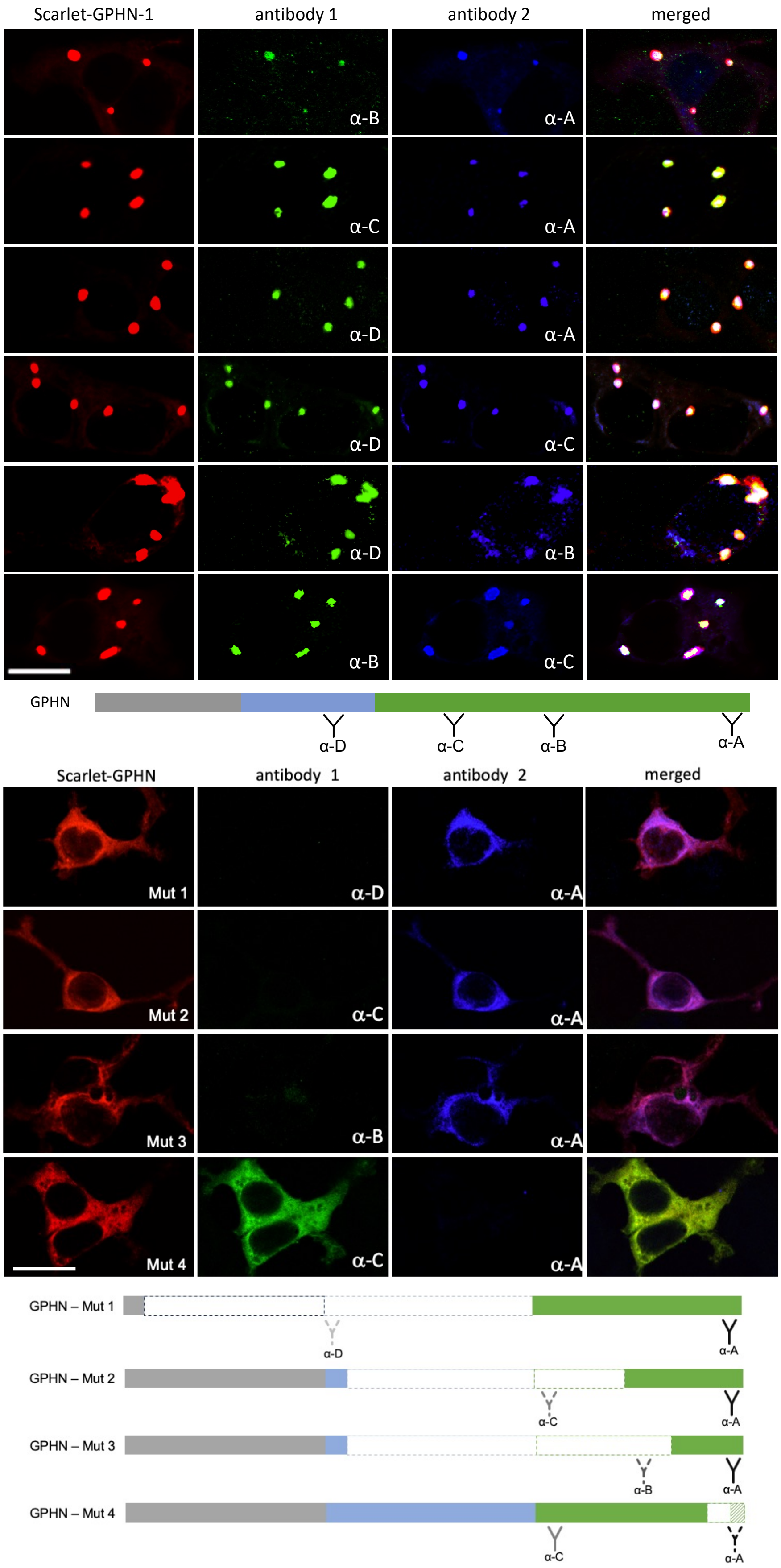

Supplementary Fig.10: Antigens recognized by GPHN antibodies.

GPHN-1 is ubiquitously labeled by all four antibody combinations (top panel). Confocal images of HEK 293 cells expressing the exogenous Scarlet-GPHN-1 protein and stained with all the possible combinations of anti-GPHN antibodies used in this study. (Scale bars: 15 µm). The epitope of each antibody is displayed on a schematic view of the GPHN primary sequence. At the bottom panel, specificity of antibodies are assayed with different GPHN isoforms. Expression of GPHN isoforms (-7, -10, -28 and -32) is detected by only one anti-GPHN antibody as expected by the presence of a single epitope on their primary sequence. Representative images were obntained from n= 3 independent experiments

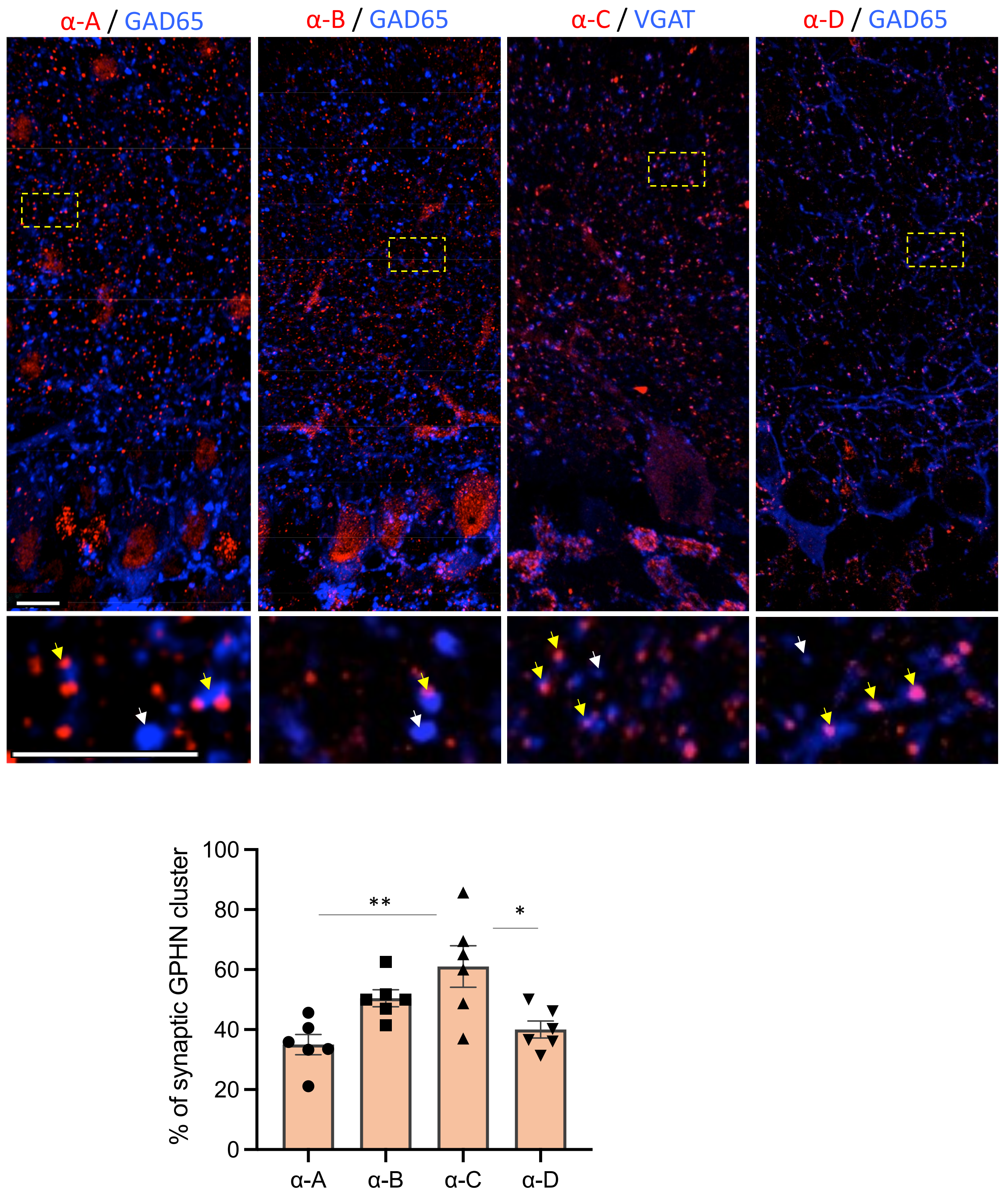

Supplementary Fig.11: *In vivo* labeling of GPHN using immunofluorescence assay.
Confocal images of cerebellar cortex slices stained with the presynaptic markers (GAD-65 or VGAT) and anti-GPHN antibodies (a-A, a-B, a-C and a-D). Presynaptic markers are labeled in blue and anti-GPHN in red. Yellow boxes indicate the region magnified below each panel. Representative images were obtained from n= 6 independent experiments. Scale bars: 15µm. Graph showing the percentage of colocalization between anti-GPHNs and the pre-synaptic marker is displayed below (n=6 mice) **P=0,0019; *P<0,05. One-way ANOVA. Source data are provided as a Source Data file.

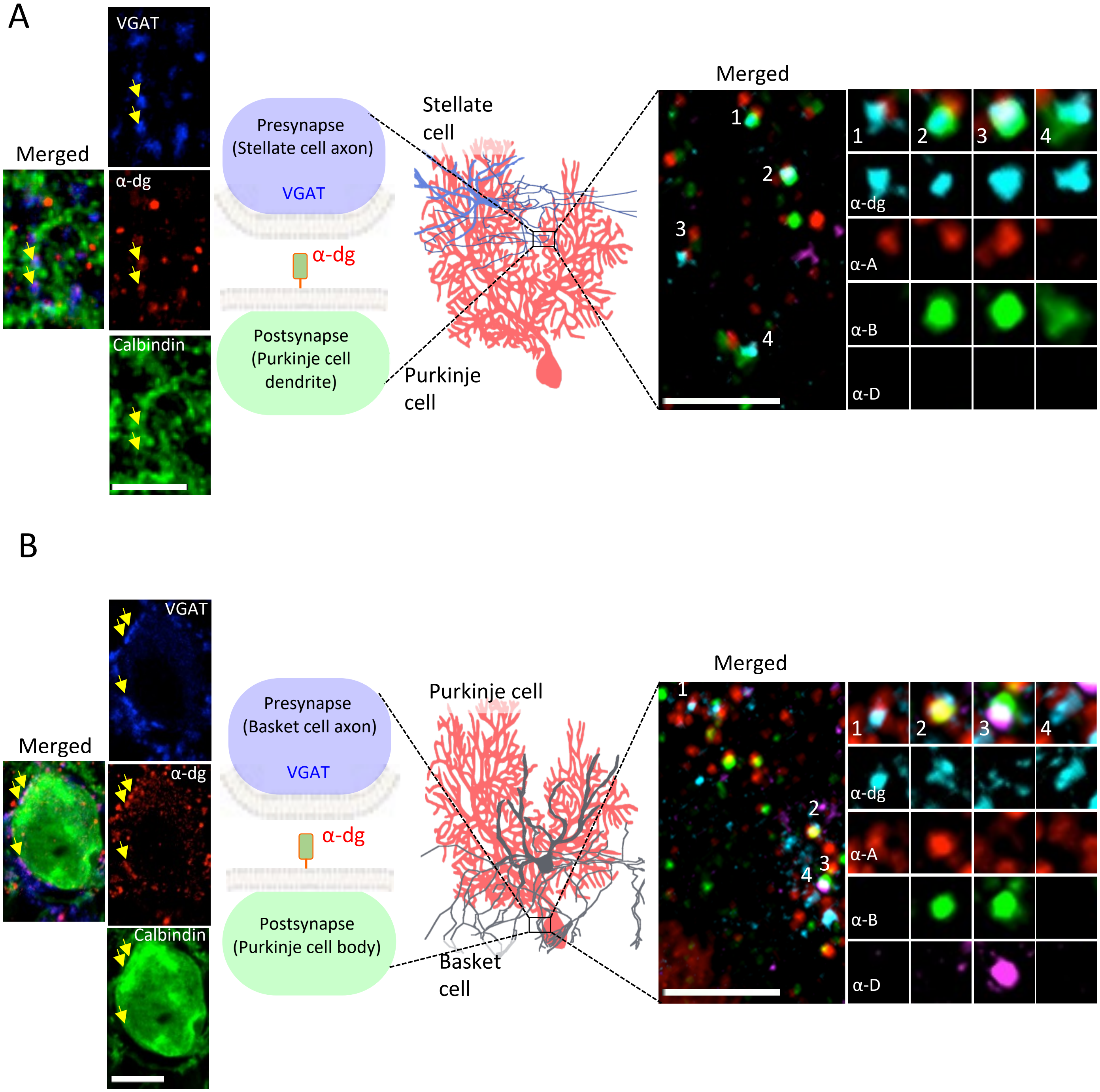

Supplementary Fig.12: *In vivo* labeling of GPHN epitopes present at inhibitory synapses localized on PC dendrites and soma.

Schematic of synapses made by stellate cell axon on PC dendrite (A; middle panel) and Basket cell on PC soma (B; middle panel). At the left panel, pre- and postsynaptic sites are detected respectively using anti-VGAT (blue) and anti-calbindin (green). Stellate cell and Basket cell inhibitory synapse made on PC are positive to α-dystroglycan (α-dg; red). At the right panel, combined GPHN epitopes labeling at α-dystroglycan positive synapse. Representative images were obntained from n= 3 independent experiments. Scale bars: 10μm.

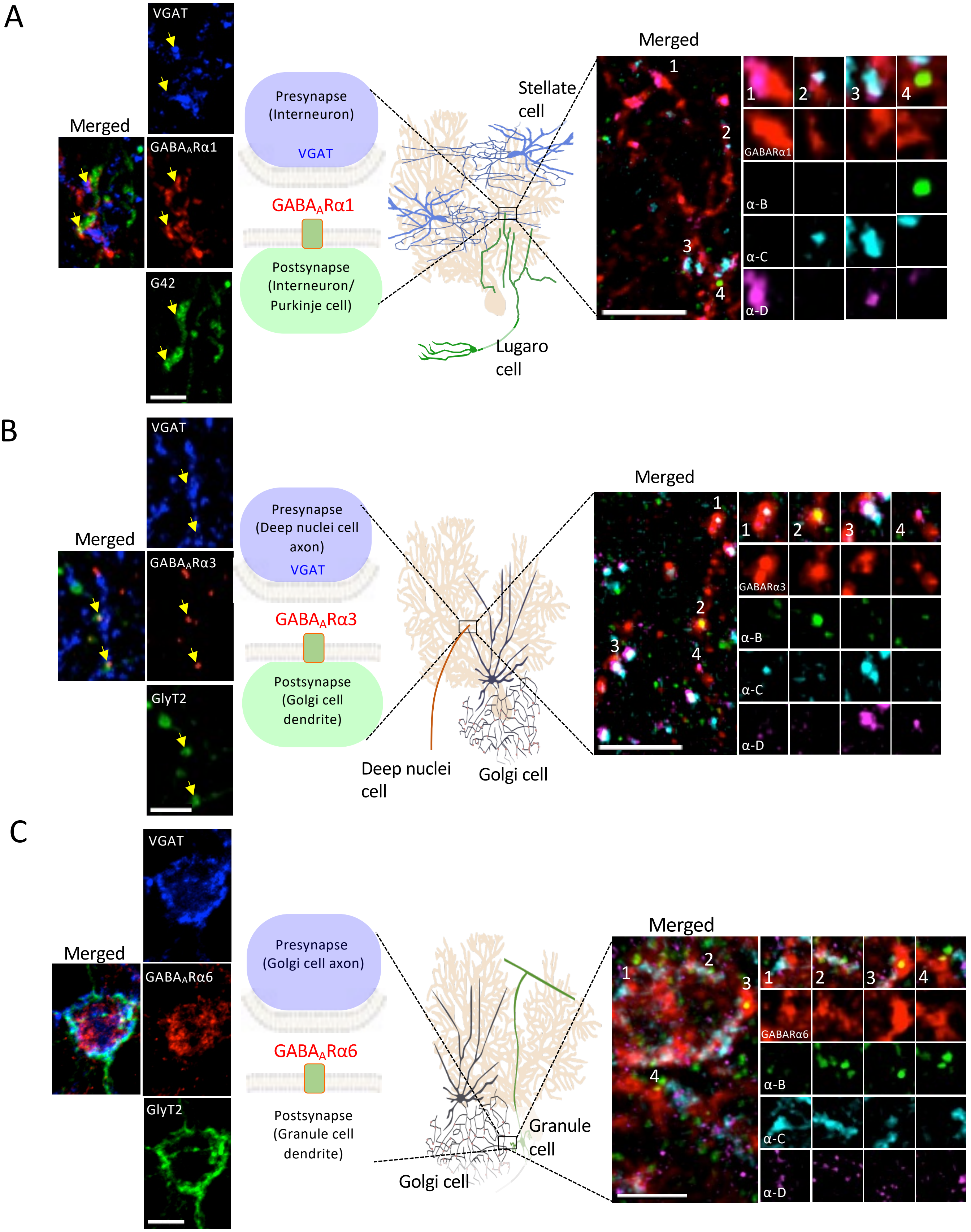

Supplementary Fig.13: Heterogeneous labeling of GPHN epitopes at inhibitory synapses containing specific GABAA Rα subunits.

Schematics in middle panels show each specific inhibitory synapse. Synapses between GABAergic interneurons and PC dendrites are positive for GABAA Rα1 (**A**). Synapses made by deep cerebellar nuclei axons on Golgi cell dendrites are positive for GABAA Rα3 (**B**). Synapses made by Golgi cell axons on Granule cell dendrites are positive for GABAA Rα6 (**C**). GFP positive inhibitory interneuron dendrites within the molecular layer are identified using the Gad67-GFP mice. Golgi Cells axon and dendrite are identified using the GlyT2-GFP mice. Representative images were obntained from n= 3 independent experiments.

Scale bars: 10µm

A

Gephyrin properties						
Clustering			Transport	Receptor recruitment		
G-Trimer	E-Dimer	Palmitoylation C212 and C284	Dlc-1/-2	$GABA_AR$	GlyR	
-	+	-	-	+	+	GPHN-5
+	+	+	+	+	+	GPHN-6
-	+/-	-	-	-	-	GPHN-7
+	+	+	+	+	+	GPHN-8
+	-	+	+	-	-	GPHN-10
+/-	+	+	+	+	+	GPHN-14
+	-	+	+	-	-	GPHN-28
+	-	+	+	-	-	GPHN-32
+	-	+	+	-	-	GPHN-42
+/-	-	+	+	-	-	GPHN-49

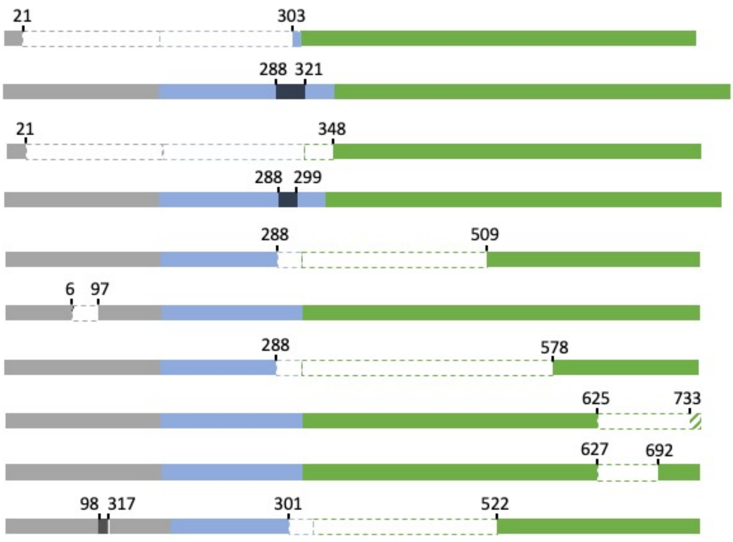

B

	Expected M.W. (kDa)
pLvx -scarlett	34,9
pLvx -scarlett-GPHN-1	109,1
pLvx -scarlett-GPHN-5	78,9
pLvx -scarlett-GPHN-6	112,8
pLvx -scarlett-GPHN-7	73,8
pLvx -scarlett-GPHN-8	111,7
pLvx -scarlett-GPHN-10	85,3
pLvx -scarlett-GPHN-14	105,7
pLvx -scarlett-GPHN-28	77,9
pLvx -scarlett-GPHN-32	96,9
pLvx -scarlett-GPHN-42	101,5
pLvx -scarlett-GPHN-49	86,8

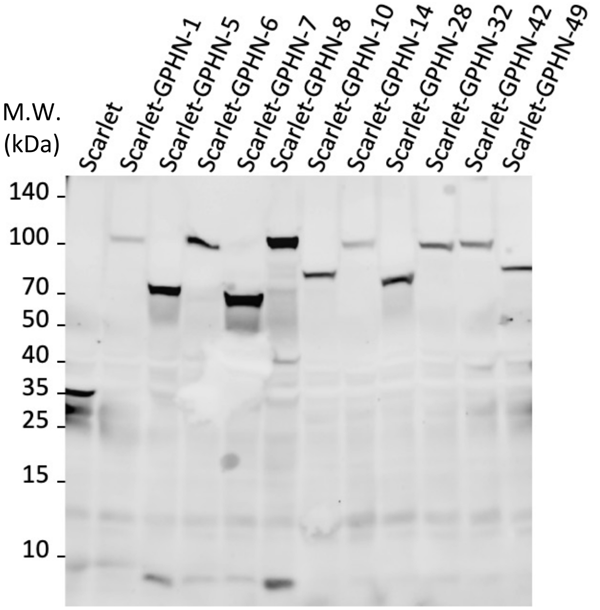

Supplementary Fig.14: Exogenous expression of several GPHN protein isoforms.
(**A**) Schematics of GPHN-5, -6, -7, -8, -10, -14, -28, -32, -42, -49 isoforms; additional domains are colored in dark blue, missing domains are shown in dashed lines and altered domains are filled with a striped motif. On the left, the table shows the prediction for GPHN isoforms to form clusters (through G-trimerization (*39*), E-dimerization (*40*)), crucial residues palmitoylation (*27*) or collybistin binding (*13*)), to interact with molecular transport (*11*), to interact with GABAARs or GlyRs (*24*), and to be anchored to the cytoskeleton (*12*). A plus sign means the sites are present in the sequence of the variant, a minus sign means the whole site is absent from the sequence. +/- means that the site is partially present in the sequence. Note that these estimations do not account for the potential conformation changes induced by the addition or removal of a peptide sequence. (**B**) At the left, a table lists the theoretical molecular weights of each GPHN isoform fused to Scarlet as well as the corresponding lentiviral constructs. At the right, the exogenous GPHN isoforms are detected by western blotting after transfection of constructs in HEK-293 cells. Exogenous factors are detected through their V5 Tag. Source data are provided as a Source Data file.

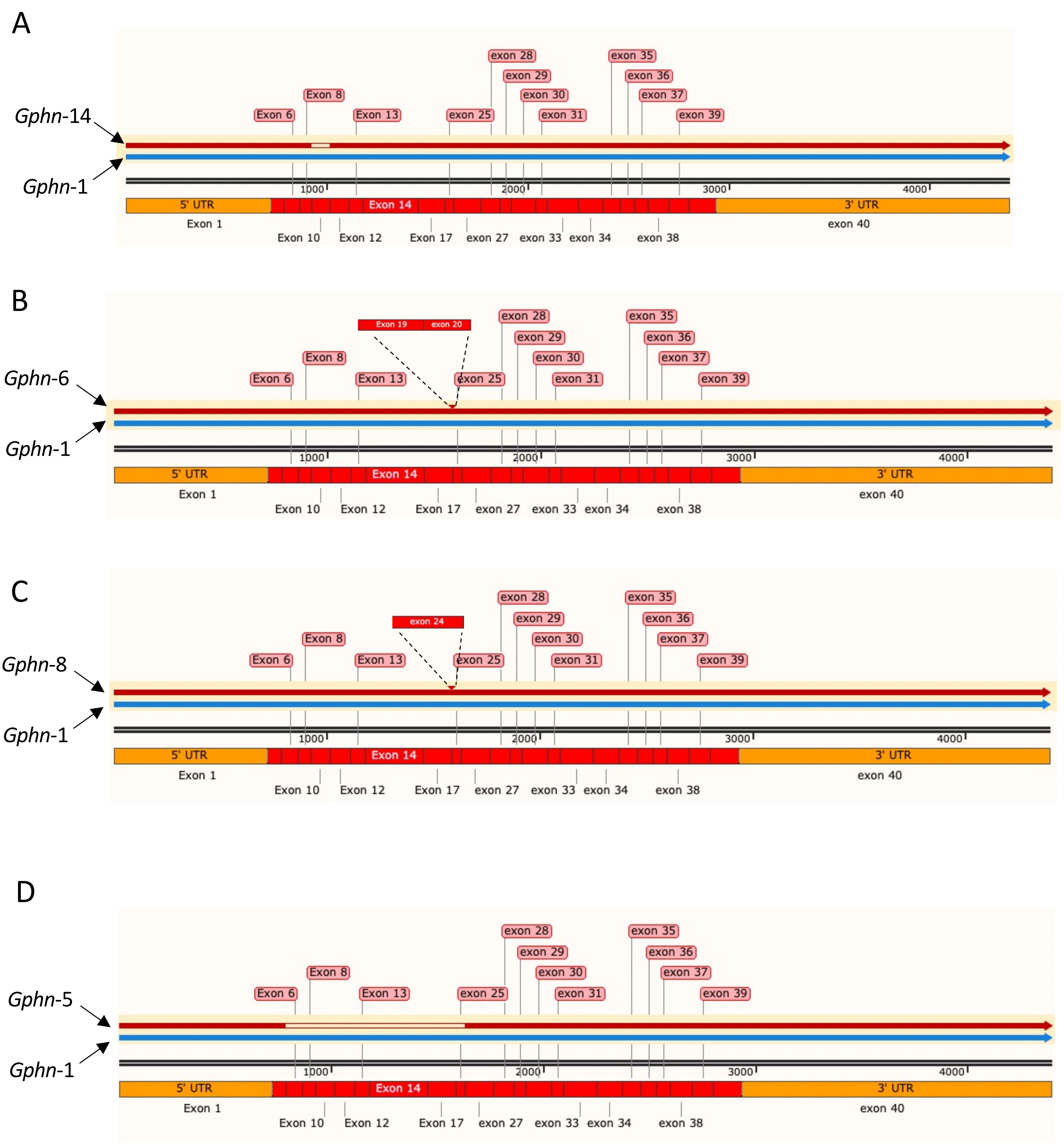

Supplementary Fig.15: Schematic representation displaying the comparison of different *Gphn* transcripts with *Gphn*-1.

A

Human	Adrenal Gland	Pooled from 62 male/female whites, ages 15-61
	Brain, Cerebellum	Pooled from 10 male/female whites, ages 22-68
	Brain, (whole)	43-year-old male white
	Fetal Brain	pooled from 59 spontaneously aborted male/female white fetuses, ages 22-33 weeks
	Fetal Liver	pooled from 63 spontaneously aborted male/female white fetuses, ages 22-40 weeks
	Heart	pooled from 3 male whites, ages 30-39
	Kidney	40-year-old female white
	Liver	51-year-old male white
	Lung	Pooled from 3 male/female whites, ages 32-61
	Placenta	Pooled from 3 whites, ages 23-30
	Prostate	Pooled from 12 whites, ages 20-58
	Salivary Gland	Pooled from 24 male/female whites, ages 16-60
	Skeletal Muscle	Pooled from 2 male/female whites, ages 43-46
	Spleen	Pooled from 15 male/female whites, ages 22-69
	Thymus	Pooled from 2 male whites, ages 18-57
	Thyroid gland	Pooled from 64 male/female whites, ages 15-61
	Trachea	Pooled from 22 male/female whites, ages 18-54
	Uterus	Pooled from 8 whites, ages 23-63
	Small intestine	Pooled from 5 male/female whites, ages 20-61
	Stomach	50-year-old male white
	Testis	NA

B

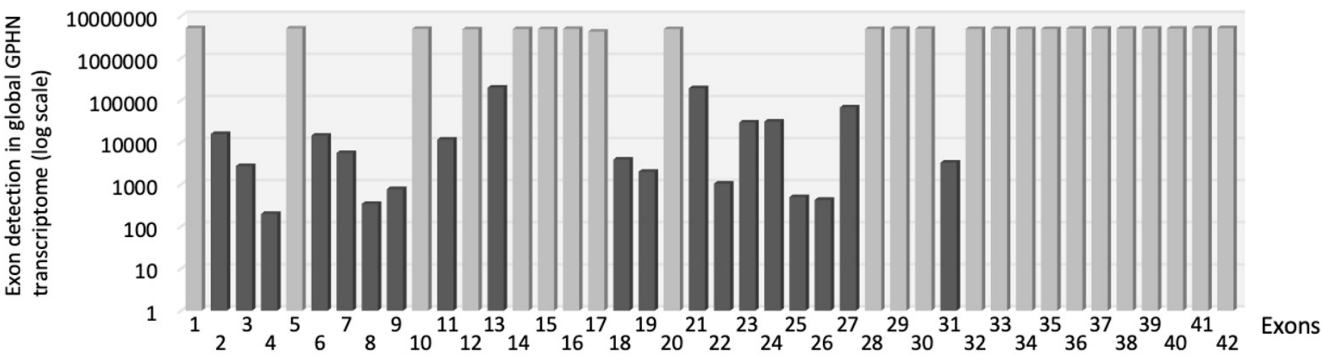

C

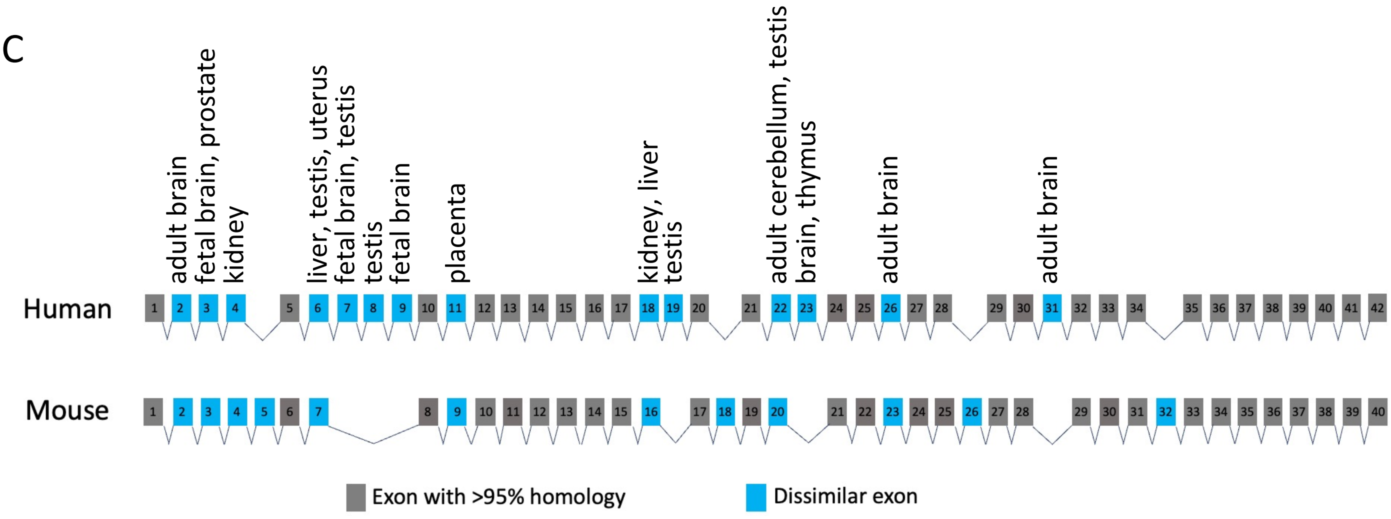

Supplementary Fig.16: *GPHN* transcriptome in 21 human tissues analyzed by long read sequencing. (**A**) Table reporting the human tissue sample (s) in which GPHN expression has been analyzed by targeted ONT sequencing. (**B**) Graph displaying how many time individual exons were detected by ONT sequencing of human tissues. Values are reported using a log scale. (**C**) Schematic representation of *GPHN* exon architecture in human and mouse, it is important to note that the comparison is made using percent nucleotide homology.

Tissues	AG	C	BW	FB	FL	H	K	L	L	P	Pr	SG	SM	Sl	S	St	Te	T	TG	tr	U	
AG	100	45,8	47,8	37,9																		
C		100	72,2	66,5	29,0	32,1	28,1	27,0	34,1	26,7	31,8	27,6	29,5	32,1	31,5	27,8	30,4	31,5	29,5	30,1	31,8	
BW			100	56,8	26,9	30,5	27,6	25,8	32,3	24,0	29,5	25,6	27,9	30,7	29,7	25,6	26,6	28,9	28,4	27,1	30,7	brain
FB				100	25,2	30,0	24,9	24,0	32,4	24,0	28,8	25,2	25,8	28,8	29,7	25,2	27,0	29,4	26,1	27,0	29,1	
FL					100																	
H						100																
K							100															
L								100														
L									100													
P										100												
Pr											100											
SG												100										
SM													100									
Sl														100								
S															100							
St																100						
Te																	100					
T																		100				
TG																			100			
tr																				100		
U																					100	

0

100

Supplementary Fig.17: Percentage of alternative *GPHN* transcripts that are common between brain and other human tissues.

Heatmap staining is used to indicate the percentage of similarity, with red meaning high overlapping (100%) and white means poor overlapping (0%).

Supplementary Materials

This file includes:

- Legends of **Supplementary Tables** 1 to 6
- Table of antibodies used in this study.

<u>**Legends of Supplementary Tables:**</u>

Supplementary Table 1: Sequences of *Gphn* alternative transcripts.
List of all mouse *Gphn* cDNAs identified in this study, note that the selected 5' primer used for PCR amplification starts 29 nucleotides after the canonical ATG. Each individual *Gphn* cDNA is named as in Supplementary Fig.2.

Supplementary Table 2: Quantification of *Gphn* exon-exon junction .
Short read sequencing datasets used to validate *Gphn* transcriptome are listed in the first sheet, while quantification of exon-exon junctions present in *Gphn* transcriptome are listed in a separated sheet.

Supplementary Table 3: Quantification of *Gphn* expression in mouse brain cells.
RNA-seq dataset used to analyze *Gphn* expression in various neuronal cells are listed in the first sheet, while in a separated sheet are quantified the expression levels of *Gphn* exons.

Supplementary Table 4: Analysis of theoretical GPHN proteome.
In the first sheet, all *Gphn* TIS provided by the ORF finder program are listed as well as those validated by Ribo-seq (yellow coloring). Gphn ORFs initiated from all TISs are listed in the "ORF finder" sheet, while those retained after TIS's validation are displayed in the "ORF retained with TIS Ribo-seq" sheet. Other processing steps are displayed in separate sheets such as the elimination of duplicated ORFs, ORFs containing PTCs. Finally, the last sheet lists the theoretical proteome of the GPHN.

Supplementary Table 5: GPHN peptides supporting new GPHN isoforms.
In the first sheet are listed all GPHN peptides identified by mass spectrometry, while in a separate sheet we displayed those validating the expression of new GPHN isoforms.

Supplementary Table 6: Human GPHN transcriptome.
List of all human *GPHN* cDNAs identified in this study (first sheet), note that the selected 5' primer used for PCR amplification starts 29 nucleotides after the canonical ATG. The frequency of exon inclusion associated with *GPHN* expression in human tissues is presented in a separated sheet.

Table of antibodies used in this study:

**Antigen**	**Host**	**Dilution**	**Supplier**	**Catalog N**	**RRID**
GAD-65	Mouse	1:500	Chemicon	MAB351R	RRID:AB_94905
Gphn (A)	Rabbit	1:1000	Abcam	ab32206	RRID:AB_2112628
Gphn (B)	Chicken	1:500	Abcam	ab136343	RRID: none
Gphn (C)	Mouse	1:500	Synaptic System	147111	RRID:AB_887719
Gphn (D)	Guinea Pig	1:250	Synaptic System	147318	RRID:AB_2661777
VGAT	Guinea Pig	1:500	Synaptic System	131004	RRID:AB_887873
α-dystroglycan	Mouse	1:500	Millipore	05-298	RRID:AB_309674
$GABA_AR\alpha1$	Rabbit	1:1000	Alomone Labs	AGA-001	RRID:AB_2039862
$GABA_AR\alpha3$	Rabbit	1:1000	Alomone Labs	AGA-003	RRID:AB_2039866
$GABA_AR\alpha6$	Rabbit	1:1000	Alomone Labs	AGA-004	RRID:AB_2039868

Calbindin	Rabbit	1:1000	Swant	CB38	RRID:AB_2721225
GFP	Chicken	1:1000	Aves Lab	GFP-1020	RRID:AB_10000240

**Secondary**	**Host**	**Dilution**	**Supplier**	**Catalog N**	**RRID**
Mouse Alexa 488	Donkey	1:500	Molecular probes	A-21202	RRID:AB_141607
Mouse Alexa 405	Goat	1:500	Molecular probes	A-31553	RRID:AB_221604
Mouse Alexa 546	Goat	1:500	Molecular probes	A-11003	RRID:AB_2534071
Rabbit Alexa 546	Goat	1:500	Molecular probes	A-11010	RRID:AB_2534077
Chiken Alexa 488	Goat	1:500	Molecular probes	A-11039	RRID:AB_142924
Guinea pig Alexa 647	Donkey	1:500	Jackson ImmunoResearch Labs	706-605	RRID:AB_2340476
