# Supplementary material for: Complex regulation of Gephyrin splicing is a determinant of inhibitory postsynaptic diversity
Source: Nat Commun. 2022 Jun 18;13:3507. doi: 10.1038/s41467-022-31264-w (PMC9206673; doi:10.1038/s41467-022-31264-w)
Supplement: Supplementary file 9 — Reporting Summary [file 41467_2022_31264_MOESM9_ESM.pdf]

nature portfolio

Corresponding author(s): Allemand Eric and Ango Fabrice

Last updated by author(s): May 3, 2022

Reporting Summary

Nature Portfolio wishes to improve the reproducibility of the work that we publish. This form provides structure for consistency and transparency in reporting. For further information on Nature Portfolio policies, see our Editorial Policies and the Editorial Policy Checklist.

Statistics

For all statistical analyses, confirm that the following items are present in the figure legend, table legend, main text, or Methods section.

n/a	Confirmed	
☐	☒	The exact sample size (*n*) for each experimental group/condition, given as a discrete number and unit of measurement
☐	☒	A statement on whether measurements were taken from distinct samples or whether the same sample was measured repeatedly
☐	☒	The statistical test(s) used AND whether they are one- or two-sided<br>*Only common tests should be described solely by name; describe more complex techniques in the Methods section.*
☐	☒	A description of all covariates tested
☐	☒	A description of any assumptions or corrections, such as tests of normality and adjustment for multiple comparisons
☐	☒	A full description of the statistical parameters including central tendency (e.g. means) or other basic estimates (e.g. regression coefficient) AND variation (e.g. standard deviation) or associated estimates of uncertainty (e.g. confidence intervals)
☐	☒	For null hypothesis testing, the test statistic (e.g. *F*, *t*, *r*) with confidence intervals, effect sizes, degrees of freedom and *P* value noted<br>*Give P values as exact values whenever suitable.*
☒	☐	For Bayesian analysis, information on the choice of priors and Markov chain Monte Carlo settings
☒	☐	For hierarchical and complex designs, identification of the appropriate level for tests and full reporting of outcomes
☒	☐	Estimates of effect sizes (e.g. Cohen's *d*, Pearson's *r*), indicating how they were calculated

Our web collection on statistics for biologists contains articles on many of the points above.

Software and code

Policy information about availability of computer code

Data collection	Confocal images were acquired using Zeiss LSM Image (V3).<br>Pacbio data were collected with an SMRT cell running on the RSII PacBio sequencer.<br>ONT data were processed with a flow cell R.9.4.1 on the MinIOn sequencing device and base calling was obtained with Guppy v3.4.1.
Data analysis	Confocal Image analysis were done on FIJI version 2.0.0-rc-69/1.52p and Soda suite software (http://icy.bioimageanalysis.org/plugin/soda-suite/). Statistical analyses were performed using GraphPad Prism (V8). For long read sequencing analysis (please see "Software policy" file for all used open source software and links.

For manuscripts utilizing custom algorithms or software that are central to the research but not yet described in published literature, software must be made available to editors and reviewers. We strongly encourage code deposition in a community repository (e.g. GitHub). See the Nature Portfolio guidelines for submitting code & software for further information.

Data

Policy information about availability of data

All manuscripts must include a data availability statement. This statement should provide the following information, where applicable:

- Accession codes, unique identifiers, or web links for publicly available datasets
- A description of any restrictions on data availability
- For clinical datasets or third party data, please ensure that the statement adheres to our policy

Sequencing data are available to ENA public database (https://www.ebi.ac.uk/ena/browser/view/):
- PacBio sequencing data: PRJEB49417
- ONT sequencing data: PRJEB49420

Field-specific reporting

Please select the one below that is the best fit for your research. If you are not sure, read the appropriate sections before making your selection.

☒ Life sciences ☐ Behavioural & social sciences ☐ Ecological, evolutionary & environmental sciences

For a reference copy of the document with all sections, see nature.com/documents/nr-reporting-summary-flat.pdf

Life sciences study design

All studies must disclose on these points even when the disclosure is negative.

Sample size	For quantitative analysis, no sample size calculation was performed. Experiments were designed so that there were at least three independent biological replicates, as an accepted standard procedure in the field. All statistical analyses were performed using GraphPad Prism 8 (GraphPad Softwares). Normality was assessed with the Shapiro-Wilk normality test. Homoscedasticity was assessed with the Barlett's test. Parametric data with the same standard deviation were analyzed by t-test, one-way ANOVA, or two-way ANOVA followed by comparison of multiple samples with Tukey post hoc analysis. Parametric data with significantly different standard deviation were analyzed by Welch's ANOVA followed by comparison of multiple samples with Dunett's T3 post hoc analysis. Non-parametric data were analyzed by the Kruskal-Wallis one-way analysis of variance on ranks followed by comparison of multiple samples with Dunn post hoc analysis. P values <0.05 were considered statistically significant. *p<0.05, **p<0.01, ***p<0.001, ****p<0.0001. ns not significant. Data are presented as mean ± SEM.
Data exclusions	No data were excluded from the analysis
Replication	Each animal experiment was carried out at least three times unless otherwise indicated. For in vitro experiment each condition was performed at least four times unless otherwise indicated, as an accepted standard procedure in the field. All attempts at replication were successful.
Randomization	Samples were randomly allocated to experimental groups in a balanced mix of male and female mice for all immunohistochemistry experiments and tissue preparation for long-read sequencing analysis.
Blinding	Two of our co-authors independently analyzed all the results of the study and were blind to the grouping.

Reporting for specific materials, systems and methods

We require information from authors about some types of materials, experimental systems and methods used in many studies. Here, indicate whether each material, system or method listed is relevant to your study. If you are not sure if a list item applies to your research, read the appropriate section before selecting a response.

Materials & experimental systems

n/a	Involved in the study	
☐	☒	Antibodies
☐	☒	Eukaryotic cell lines
☒	☐	Palaeontology and archaeology
☐	☒	Animals and other organisms
☐	☒	Human research participants
☒	☐	Clinical data
☒	☐	Dual use research of concern

Methods

n/a	Involved in the study	
☒	☐	ChIP-seq
☒	☐	Flow cytometry
☒	☐	MRI-based neuroimaging

Antibodies

Antibodies used	Antibodies used in this study:<br>- Antigen: GAD-65, Host: Mouse, Dilution: 1:500, Supplier: Chemicon, Catalog N: MAB351R, RRID : AB_94905<br>- Antigen: Gphn (A), Host: Rabbit, Dilution: 1:1000, Supplier: Abcam, Catalog N: ab32206, RRID : AB_2112628<br>- Antigen: Gphn (B), Host: Chicken, Dilution: 1:500, Supplier: Abcam, Catalog N: ab136343, RRID : none<br>- Antigen: Gphn (C), Host: Mouse, Dilution: 1:500, Supplier: Synaptic System, Catalog N: 147111, RRID : AB_887719<br>- Antigen: Gphn (D), Host: Guinea Pig, Dilution: 1:250, Supplier: Synaptic System, Catalog N: 147318, RRID : AB_2661777<br>- Antigen: VGAT, Host: Guinea Pig, Dilution: 1:500, Supplier: Synaptic System, Catalog N: 131004, RRID : AB_887873<br>- Antigen: α-dystroglycan, Host: Mouse, Dilution: 1:500, Supplier: Millipore, Catalog N: 05-298, RRID : AB_309674<br>- Antigen: GABAARα1, Host: Rabbit, Dilution: 1:1000, Supplier: Alomone Labs, Catalog N: AGA-001, RRID : AB_2039862<br>- Antigen: GABAARα3, Host: Rabbit, Dilution: 1:1000, Supplier: Alomone Labs, Catalog N: AGA-003, RRID : AB_2039866<br>- Antigen: GABAARα6, Host: Rabbit, Dilution: 1:1000, Supplier: Alomone Labs, Catalog N: AGA-004, RRID : AB_2039868

- Antigen: Calbindin, Host: Rabbit, Dilution: 1:1000, Supplier: Swant, Catalog N: CB38, RRID : AB_2721225
- Antigen: GFP, Host: Chicken, Dilution: 1:1000, Supplier: Aves Lab, Catalog N: GFP-1020, RRID : AB_10000240
- Antigen: Mouse Alexa 488, Host: Donkey, Dilution: 1:500, Supplier: Molecular probes, Catalog N: A-21202, RRID : AB_141607
- Antigen: Mouse Alexa 405, Host: Goat, Dilution: 1:500, Supplier: Molecular probes, Catalog N: A-31553, RRID : AB_221604
- Antigen: Mouse Alexa 546, Host: Goat, Dilution: 1:500, Supplier: Molecular probes, Catalog N: A-11003, RRID : AB_2534071
- Antigen: Rabbit Alexa 546, Host: Goat, Dilution: 1:500, Supplier: Molecular probes, Catalog N: A-11010, RRID : AB_2534077
- Antigen: Chiken Alexa 488, Host: Goat, Dilution: 1:500, Supplier: Molecular probes, Catalog N: A-11039, RRID : AB_142924
- Antigen: Guinea pig Alexa 647, Host: Donkey, Dilution: 1:500, Supplier: Jackson ImmunoResearch Labs, Catalog N: 706-605, RRID : AB_2340476

Validation

The antibodies used in the study were all purchased from reputable commercial sources. All antibodies are widely used and validated by the providers or previous publications. Below are the manufacturer's links to the antibody information and relevant citations.
GAD-65 (MAB351R, Chemicon) https://www.merckmillipore.com/FR/fr/product/Anti-Glutamate-Decarboxylase-Antibody-65-kDa-isoform-clone-GAD-6,MM_NF-MAB351R?CatalogCategoryID=&ReferrerURL=https%3A%2F%2Fwww.google.com%2F#anchor_REF
GPHN (ab32206, Abcam) https://www.abcam.com/ab32206.pdf?
GPHN (ab136343, Abcam) https://www.abcam.com/ab136343.pdf?
GPHN (147111, SYSY) https://sysy.com/product/147111
GPHN (147318, SYSY) https://sysy.com/product/147318#list
VGAT (131004, SYSY) https://sysy.com/product/131004#list
α-dystroglycan (05-298, Millipore) https://www.merckmillipore.com/FR/fr/product/Anti-Dystroglycan-Antibody-clone-VIA4-1,MM_NF-05-298
GABAARα1 (AGA-001, Alomone Labs) https://www.alomone.com/p/anti-gabaa-1-receptor-extracellular/AGA-001
GABAARα3 (AGA-003, Alomone Labs) https://www.alomone.com/p/anti-gabaa-3-receptor-extracellular/AGA-003
GABAARα6(AGA-004, Alomone Labs) https://www.alomone.com/p/anti-gabaa-6-receptor-extracellular/AGA-004
Calbindin (CB38, Swant) https://www.swant.com/pdfs/CB38_Rabbit%20anti%20Calbindin%20D-28k%20500%20ul.pdf
GFP (GFP-1020, Aves Lab) https://www.aveslabs.com/products/anti-green-fluorescent-protein-antibody-gfp

Eukaryotic cell lines

Policy information about cell lines

Cell line source(s)

293 [HEK-293]

CRL-1573™
HEK 293 from ATCC (CRL-1573)

Authentication

None of the cell line used were authenticated

Mycoplasma contamination

Line were tested negative for micoplasma contamination

Commonly misidentified lines
(See ICLAC register)

Nothing Found

Animals and other organisms

Policy information about studies involving animals; ARRIVE guidelines recommended for reporting animal research

Laboratory animals

Male and female C57BL/6 mice of 8–10 weeks (weighing 20–25 g) were used for animal experiments. For developmental point analysis, pups aged from P0 to P21 were collected from C57BL/6 mice breeding. WT mice were purchased from Charles River Laboratories. Primary hippocampal neurons were dissected from fetal WT C57BL/6 mice

Wild animals

No wild animals

Field-collected samples

the study did not involved samples collected from the field.

Ethics oversight

We followed the European and national regulations for the care and use of animals in order to protect vertebrate animals for experimental and other scientific purposes (Directive 86/609).

Note that full information on the approval of the study protocol must also be provided in the manuscript.

Human research participants

Policy information about studies involving human research participants

Population characteristics

RNA from human tissues were commecially bought to the following reference (Clontech #636533 and #636643). The origins of samples are described bellow:
Adrenal Gland (Pooled from 62 male/female caucasians, ages 15-61)
Brain, Cerebellum (Pooled from 10 male/female caucasians, ages 22-68)
Brain, whole (43-year-old male caucasian)
Fetal Brain (pooled from 59 spontaneously aborted male/female caucasian fetuses, ages 22-33 weeks)
Fetal Liver (pooled from 63 spontaneously aborted male/female caucasian fetuses, ages 22-40 weeks)

Heart (pooled from 3 male caucasians, ages 30-39)
Kidney (40-year-old female Caucasian)
Liver (51-year-old male caucasian)
Lung (Pooled from 3 male/female caucasians, ages 32-61)
Placenta (Pooled from 3 caucasians, ages 23-30)
Prostate (Pooled from 12 caucasians, ages 20-58)
Salivary (Gland Pooled from 24 male/female caucasians, ages 16-60)
Skeletal (Muscle Pooled from 2 male/female caucasians, ages 43-46)
Spleen (Pooled from 15 male/female caucasians, ages 22-69)
Thymus (Pooled from 2 male caucasians, ages 18-57)
Thyroid gland (Pooled from 64 male/female caucasians, ages 15-61)
Trachea (Pooled from 22 male/female caucasians, ages 18-54)
Uterus (Pooled from 8 caucasians, ages 23-63)
Small intestine (Pooled from 5 male/female caucasians, ages 20-61)
Stomach (50-year-old male caucasian)
Testis (N/A)

Recruitment	N/A
Ethics oversight	N/A

Note that full information on the approval of the study protocol must also be provided in the manuscript.
